# Bone Laser Patterning to Decipher Cell Organization

**DOI:** 10.3390/bioengineering10020155

**Published:** 2023-01-24

**Authors:** Nicolas Touya, Samy Al-Bourgol, Théo Désigaux, Olivia Kérourédan, Laura Gemini, Rainer Kling, Raphaël Devillard

**Affiliations:** 1 Univ. Bordeaux, INSERM, BIOTIS, U1026, F-33000 Bordeaux, France; 2 ALPhANOV, Rue François Mitterrand, 33400 Talence, France; 3 Faculty of Dentistry, University of Bordeaux, 146 rue Léo Saignat, 33076 Bordeaux, France; 4 Pôle de Médecine et Chirurgie Bucco-Dentaire, CHU de Bordeaux, Place Amélie Raba Léon, 33076 Bordeaux, France

**Keywords:** tissue engineering, bone, laser, femtosecond, patterning, direct

## Abstract

The laser patterning of implant materials for bone tissue engineering purposes has proven to be a promising technique for controlling cell properties such as adhesion or differentiation, resulting in enhanced osteointegration. However, the possibility of patterning the bone tissue side interface to generate microstructure effects has never been investigated. In the present study, three different laser-generated patterns were machined on the bone surface with the aim of identifying the best surface morphology compatible with osteogenic-related cell recolonization. The laser-patterned bone tissue was characterized by scanning electron microscopy and confocal microscopy in order to obtain a comprehensive picture of the bone surface morphology. The cortical bone patterning impact on cell compatibility and cytoskeleton rearrangement on the patterned surfaces was assessed using Stromal Cells from the Apical Papilla (SCAPs). The results indicated that laser machining had no detrimental effect on consecutively seeded cell metabolism. Orientation assays revealed that patterns with larger hatch distances were correlated with higher cell cytoskeletal conformation to the laser-machined patterns. To the best of our knowledge, this study is the first to consider and evaluate bone as a biological interface that can be engineered for improvement. Further investigations should focus on the in vivo implications of this direct patterning.

## 1. Introduction

In recent years, surface modification has become a critical aspect of tissue engineering [1,2] to improve the osteointegration of implants and influence cell fate [3]. Investigations have been conducted on various synthetic materials designed to substitute bone tissue as prostheses, such as ceramics [4,5,6], metals [7,8,9,10], and polymers [11]. An in vivo study outlined the strategic importance of modifying the topography of implants with lasers by showing a stronger bone-to-implant bond [12]. Femtosecond (fs) lasers allow for the biological response tailoring of surface morphology by controlling the surface pattern geometry down to a 100 s-nm scale [13,14,15] thanks to their unique characteristics. Indeed, Ultra Short Pulses (USP) allow for a quasi-non-thermal interaction with materials and the generation of interference-based physical phenomena [16]. For example, Carvalho et al. [13] reported the increased metabolic activity of MC3T3 (osteoblastic-related cells) cultured on Alumina-Toughened Zirconia (ATZ), where grid-structures or groove-like structures were generated via fs laser machining. Gnilitskyi et al. [14] demonstrated the higher ratio of HDFa (fibroblast cells) growth on both laser-nanostructured titanium alloy and zirconium, showing the predominant role of surface morphology over the material type for these types of biological responses. A similar result was achieved by Lee et al. [15], where a variation in the Saos-2 (osteoblastic-related cells) response and surface biocompatibility was observed down to a few 100 s-nm pattern size differences.

Due to their unique characteristics, these types of laser sources are currently being considered for use in the ablation of bone tissue for surgical applications, where it is crucial (i) to preserve the healthy status of the tissue after treatment to improve its biological response, such as implant integration, and (ii) to achieve high-resolution modification of the tissue, such as in specific maxillofacial surgery approaches. A few reports have showed the avoidance of detrimental thermal effects on bone tissue after USP laser ablation, cutting, and drilling [17,18]. Detrimental effects linked to thermal accumulation, such as the presence of microparticles of melted tissue and calcined tissue, were reported to be visible on the bone tissue down to the nanosecond (ns) regime of laser–tissue interaction and to become negligible for laser pulse durations below a few picoseconds (ps) and optimized laser process parameters [19].

In this context, while a few reports on the laser patterning of synthetic hydroxyapatite are available [20], to the best of our knowledge, no reports are available in the literature dedicated to direct bone patterning using fs lasers. This new aspect of the tissue interface could promote the osteointegration of grafts and/or synergize with the superior osteointegration of laser-treated implants. In this study, the patterning of bone to potentialize current and future biomedical applications was evaluated in an in vitro model as a first step toward the possible development of next-generation strategies for the engineering of the bone/implant interface.

## 2. Materials and Methods

### 2.1. Bone Tissue Supply

Porcine femurs were collected from the same place (Casino supermarché, Talence, France) and labeled with the age and sex of the animals. All the animals from which the femurs were retrieved were intended for human consumption, in accordance with French and European regulations regarding breeding, slaughter, and hygiene-sanitation in the meat industry.

**Figure 1:**
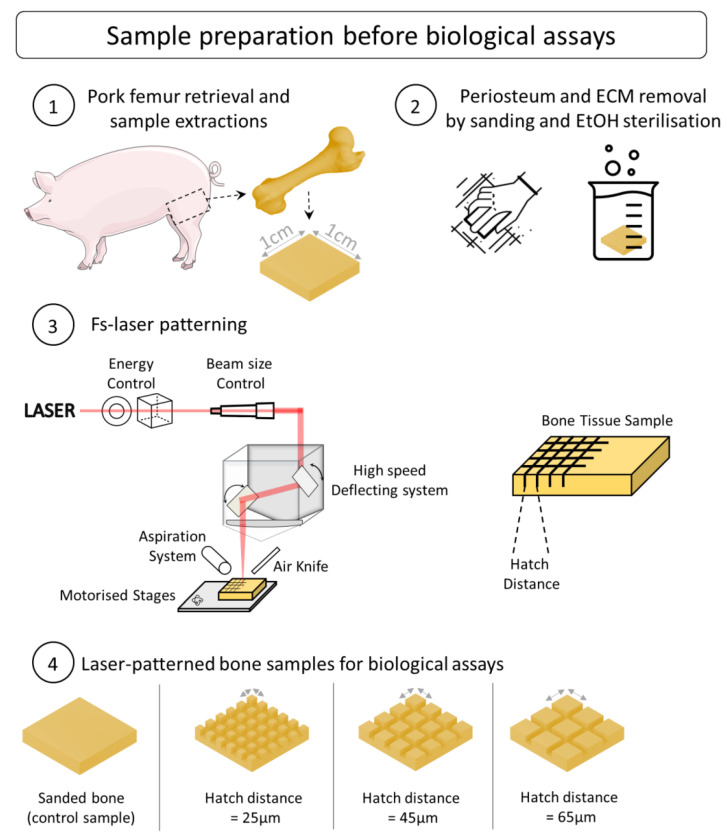
Illustrated experimental approach and protocols for preparation of bone samples before biological assays.

The bones were stored in a freezer at −20 °C. To prepare the bone tissue for laser treatment, it was left defrosting at room temperature in a laminar air-flow protection system (Thermo Scientific HERAsafe KS12, Bordeaux, France). The proximal and distal epiphysis, as well as the metaphysis parts, were then mechanically removed in order to obtain a clean cut of the diaphysis. The soft and fatty tissue, including the bone marrow, were removed using a scalpel. Finally, several samples (approx. 0.5 L × 0.4 L × 0.3 h cm) were obtained (Figure 1) from the diaphysis using a diamond blade for ease of handling during processing and characterization. All the bone samples were sanded using a mechanical sanding system (EcoMet 300, Buehler, IL, USA) to generate similar surfaces and diminish the natural topography variation and inter-sample variability. Their thickness, width, and height were measured after sanding to ensure that all the sample dimensions were homogeneous. To reproduce the operative procedure, all the samples were sterilized before laser processing (via successive ethanol baths, 24 h at 90% concentration and 4 h at 100% concentration, room temperature).

### 2.2. Laser-Assisted Patterning of Bone

A Satsuma HP2 laser (Amplitude, Talence, France) was employed for all the tests. The laser ran at a central wavelength of 1030 nm, with a maximum average power of 40 W and a pulse duration of about 350 fs. The Gaussian laser beam (TEM_00_, M^2^ < 1.2) was first sent through a half-wave plate coupled with a polarizing cube for the fine control of the laser pulse energy and then through a telescope system to adjust the beam size before the focusing optics (Figure 1). The laser beam was deflected onto the sample by a galvanometric scanning head (ScanCUBE, ScanLAB, Munich, Germany) and focused with a 100 mm f-theta lens on the sample surface. The focused laser beam had a circular focal spot with a 20 μm diameter, which was determined with a beam profiler (WinCam Dataray, CA, USA) as the full width half maximum. For all the tests, the following process parameters were kept constant: laser repetition rate of 500 kHz, average power of 5.28 W, and scanning speed of 4 m/s. The laser fluence was fixed at 3.36 J/cm^2^, which corresponded to a laser intensity of about 1 × 10^13^ W/cm^2^ and a peak power of 32 MW. This regime of interaction is typical for fs laser processing where industrial fs laser sources are employed (laser pulse energy ~ 10 s µJ at repetition rates ~ 100 s kHz and pulse duration ~ 100 s fs). Previous work on the ablation of bone tissue using ultrafast lasers has demonstrated the possibility of achieving bone tissue ablation with laser intensities well below 10^14^ W/cm^2^ [21,22,23]. The selection of the processing parameters was performed based on the results from the same group [16], which showed that in the selected process window, it was possible to ablate the bone tissue without important thermal damage. A cross-hatch pattern was generated on the sample surface, with the hatch distance being defined as the distance between two consecutive lines. Three different values of hatch distance were selected: h = 25, 45, and 65 μm. In order to achieve reliable and reproductible ablation depth values, the cross-hatch pattern was repeated 10 times for each test. An aspiration system and an air knife were employed to efficiently remove the bone debris and particles during the laser ablation. The samples were placed on motorized stages for automated positioning before the laser processing.

Profilometric assays were performed with Confomap v8 software according to methods described in ISO 5436.

### 2.3. Cell Culture

The human Stromal Cells from the Apical Papilla (SCAPs) used for the experiments were taken from a batch of cells already characterized [24]. The cells were amplified and cultured under 21% O_2_. Briefly, the cells were derived from germs of third molars obtained from young patients at the Service de Médecine bucco dentaire du Centre Hospitalier Universitaire de Bordeaux. All the experimental protocols were conducted in accordance with the guidelines and regulations under the ministerial approbation regarding French law (‘DC-2008-412′; convention INSERM-CHU de Bordeaux). All the patients gave explicit consent to have their samples used for research purposes. If a patient was under majority status regarding age, additional consent from a parent and/or legal guardian was requested. The samples were treated anonymously. The freshly extracted teeth were placed in Minimum Essential Medium Alpha (α-MEM, Gibco, Paisley, Scotland, UK) supplemented with 20% FBS and penicillin (100 U mL^−1^)/streptomycin (100 μg mL^−1^) (Life Technologies, SAS). The cells were extracted according to a previously described method [25]. The dental apical papillae were digested in a mixture of 3 mg mL^−1^ collagenase (Sigma-Aldrich) and 4 mg mL^−1^ dispase (Sigma-Aldrich) for 1 h at 37 °C. The obtained suspension was sifted through a 40 μm hole-size nylon sieve (BD Biosciences, France), and the cells were then seeded in a 75 cm^2^ culture flask (37 °C, 5% CO_2_). Any non-adherent cells were removed after 2 days, and the medium was changed thrice a week until the cells reached 95% confluence. All the SCAPs were amplified or cultured in tissue culture flasks in α-MEM supplemented with 10% FBS in a controlled atmosphere (5% CO_2_, 95% Relative Humidity, 37 °C). All the cells were cultured and used for experimentations with a maximum passage number under 5.

### 2.4. Cell Preparation

The cells were detached from the polystyrene tissue culture surface using a solution of trypsin-EDTA (0.05% 1X Gibco, Abingdon, UK). The cells were suspended in their respective culture media. The cell suspension concentration was adjusted to 10 million cells.mL^−1^ in accordance with a previously described protocol [26].

### 2.5. Metabolic Assay

After being washed in culture medium, all the samples were placed over 2% *w*/*v* agarose coated wells. An identical number of non-labeled cells (2000 cells mm^−2^) were seeded on the prepared bone samples. As a control, some cells were seeded over uncoated wells. The media were renewed every two days, and observations were made at days 1, 3, 5, and 7. Briefly, AlamarBlue™ [27] (0.1 mg mL^−1^, Sigma-Aldrich, France) was diluted at 1:15 (*v*/*v*) in standard culture medium and placed with the cells for 2 h at 37 °C. The supernatants were retrieved and transferred to a microplate, while the wells containing the cells were rinsed, refilled with standard culture medium, and placed back in the incubator. The plate reading was performed with a Varioskan Lux (Thermo Scientific, Waltham, MA, USA) spectrophotometer (λ_ex_ 530 nm and λ_em_ 590 nm).

### 2.6. Qualitative Observations via Confocal Imaging and Scanning Electron Microscopy

After the last metabolic assay on day 7, all the samples were rinsed in phosphate-buffered saline (PBS) and fixed in paraformaldehyde (4% *w*/*v*) overnight. The surfaces where cells were seeded were covered with a permeabilization mix (BSA 2% [*w*/*v*], Triton 0.1% *v*/*v*) for 2 h at 37 °C. A staining mix composed of DAPI (0.1% *v*/*v*) and Alexa546-conjugated phalloidin (0.5% *v*/*v*) in a permeabilization mix was then applied for 2 h at 37 °C. All the samples were rinsed multiple times in PBS solution before the confocal microscopy acquisition (Leica TCS SPE). ImageJ [28] and Imaris (Oxford Instruments plc., Abingdon, UK) software were used to perform the qualitative analysis of the acquisitions.

The samples were dried in consecutive ethanol baths (30%, 50%, 70%, 90%, and 99%) with 1 h soaking steps and then coated with gold using a sputter coater (EMSCOPE SC500, Elexience, Verrieres Le Buisson Cedex, France) before the SEM (TM4000+ Hitachi, Tokyo, Japan) observations.

### 2.7. Cytoskeleton Orientation Assays

To obtain a quantitative analysis of the cytoskeleton rearrangement, the Fiji plugin Directionality tool (freely available on GitHub https://github.com/fiji/Directionality, accessed on 4 May 2022) was used. First, hyperstacks of the height-through acquisitions were constructed. The sample matrix opacity-mediated autofluorescence was found to be equal in all the conditions observed.

All the pictures were manually aligned according to the laser-carved patterns so that the trenches would be parallel (0° and 180°) and perpendicular (90°) to the Fast Fourier Transform (FFT) plan.

As SCAPs physiologically self-organize in a spindle shape with a narrowing space between the cells as the population grows, an increase in the signal was expected to be reached at a certain angle. The control samples were then oriented with respect to this physiological feature, at 0° and 180° with respect to the FFT plan. To refine the orientation results, 180 steps of FFT were performed from −45° to 134°.

All the results were then gathered and analyzed using GraphPad Prism version 8.0.0 software (GraphPad Software, San Diego, CA, USA) to generate the FFT heatmaps. Circular histogram frequency plots (with a frequency proportional to the area of each bar) were obtained through the adaptation of an open-source code from matplotlib.org (the version used for this article is available upon request and free of use).

### 2.8. Statistical Analysis

All the tests were performed using GraphPad Prism version 8.0.0 software (GraphPad Software, San Diego, CA, USA). Non-parametric Kruskal–Wallis tests were selected, as the sample size was unsuitable for performing normality tests. The H0 hypothesis was rejected when the *p*-value was < 0.05, and Tukey’s post-hoc test was performed when H1 was accepted.

## 3. Results

### 3.1. Laser Patterning Profilometry

**Figure 2:**
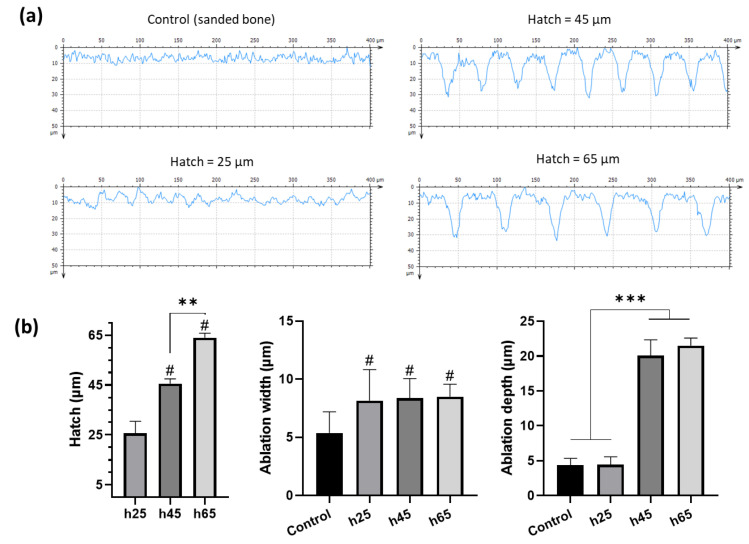
Profilometric analysis of the control (sanded bone) and laser-patterned samples. Examples of each condition surface morphology: (**a**) acquired with Confomap ST 8.2 software; and (**b**) statistical tests of the patterning physical parameters (Kruskal–Wallis, * *p* < 0.05, ** *p* < 0.01, ***p < 0.001, # = *** compared to leftmost bar. N = 3, n ≥ 12). Controls were sanded bone not patterned with a laser. Numbers are displayed as intrinsic sample surface morphology.

Profilometry measurements of the samples were performed (Figure 2) to evaluate the inter-condition differences regarding the impact of the laser patterning (Figure 2a). For the profiles obtained on samples treated with a hatch distance of 25 μm, the microroughness was adjusted by applying band-pass filtering with a cut-off wavelength of 5 μm in order to have more reliable data. It is important to underline that control samples (sanded bones) were used as a reference, and the values were presented to show the intrinsic surface morphology after sanding.

Descriptive statistics concerning all the obtained measurements are reported in Table 1, and they were used to perform the statistical analysis (Figure 2b).

The qualitative appreciation of the different profiles (Figure 2a) underlines the differences between the defined patterns of the laser-ablated bones, especially h45 and h65 µm, compared with the control.

The measured hatch distances (Figure 2b, left) were consistent with the experimental procedure and had high reproducibility. The width of the ablation (Figure 2b, center) remained constant regardless of the hatch distance used, with a value of ~8.3 µm. However, the ablation depth results (Figure 2b, right) highlighted that a 25 µm hatching was ablating a large area of bone. As a result, the surface morphology did not differ significantly from that of the control samples. With an increase of the hatch, the ablation depth was found to generate around a 21 μm deep pattern through the cortical bone.

### 3.2. Laser Machining Impact on Recolonizing Cell Survival

**Figure 3:**
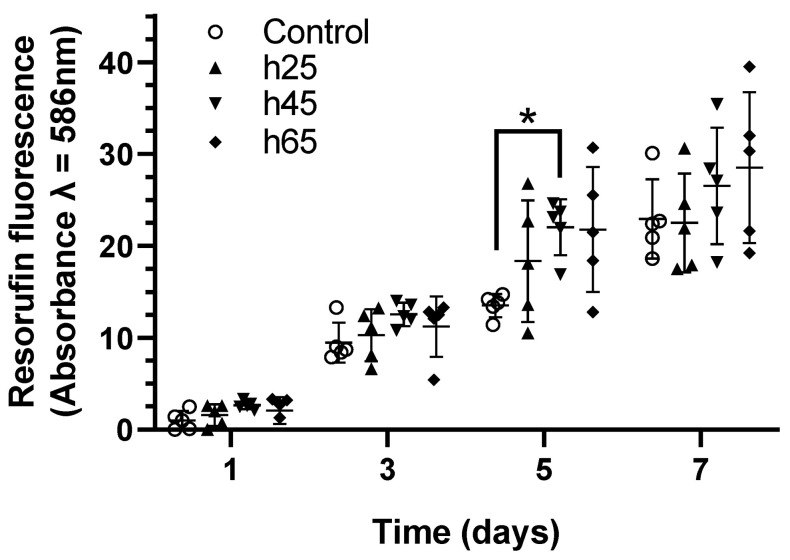
Metabolic activity of SCAPs seeded over different laser-treated samples compared with sanded bone. A total of 2000 cells mm^−2^ were seeded at day 0, and measurements were performed on days 1, 3, 5, and 7. The normalized resorufin-measured absorbance shows the cell viability and growth indirectly via the NADH dehydrogenase activity through time. n = 5, Kruskal–Wallis test *: *p* < 0.05.

The levels of resazurin reduction in the SCAPs grown on laser-patterned bones, regardless of the pattern geometries, were similar to the behavior of the SCAPs grown on the control samples (Figure 3). Over time, the SCAPs increased their metabolic activity. In all the experiments, the absorbances from a 25 µm hatch distance were observed to be close to the controls, while a trend toward higher absorbances was observed for the samples with 45 µm and 65 µm hatch distances. On day 5, the fluorescence signal from the samples patterned with a 45 µm hatch distance was found to be significantly higher than the controls. Taken together, these results suggest that laser-patterning bone tissue as a pre-treatment for cell growth is compatible with cell metabolism and suitable for SCAPs.

### 3.3. Laser Machining Impact on Cell Orientation

**Figure 4:**
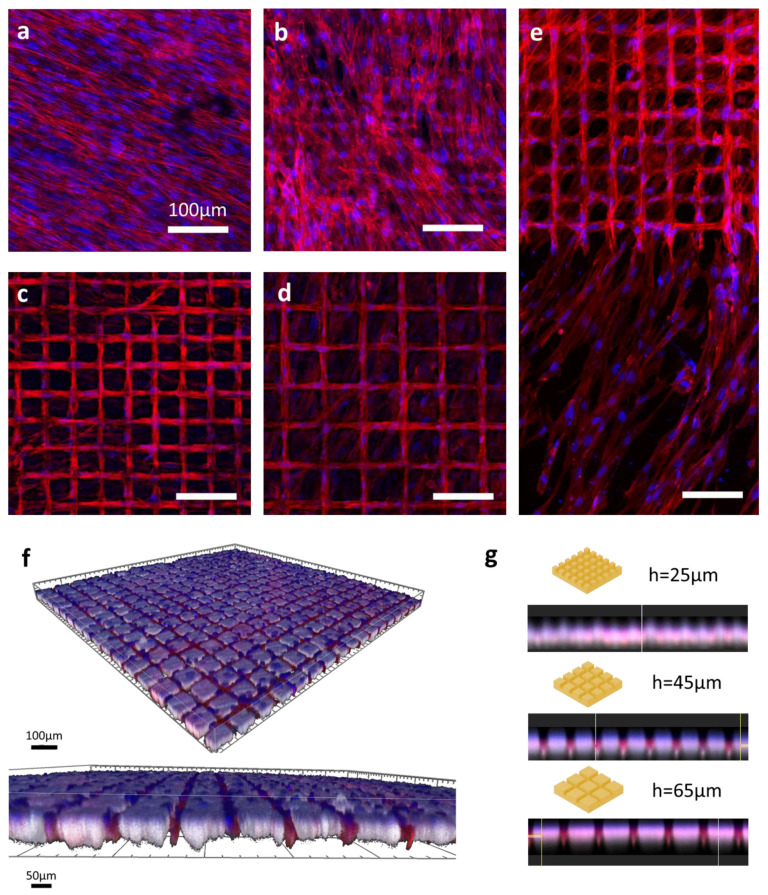
Confocal microscopy imaging of SCAPs seeded over laser-patterned bones. F-actin (red) and nucleus (blue) structures were respectively stained with Alexa 546-Phalloidin and DAPI. Images were captured using a confocal Leica TCS SPE microscope and assembled on ImageJ software. Images represent the control sample (**a**) and samples patterned with hatch distances of 25 µm (**b**), 45 µm (**c**), and 65 µm (**d**). The right-sided panel (**e**) shows the edge zone between the laser-patterned area and untreated bone on an h45 µm sample. Unspecified scale bars = 100 μm. Lower panels depict the 3D reconstruction of confocal z stack acquisitions with an illustration of a sample with a hatch distance of 65 µm (**f**) and mean orthogonal views (**g**) of samples with 25, 45, and 65 µm hatch distances.

The images obtained via confocal microscopy after 7 days of culture (Figure 4) gave the first qualitative information on the cell morphology with respect to the specific laser-generated patterns.

The cells seeded over the control (sanded bone) samples (Figure 4a) had a very stretched spindle shape. On the samples patterned with a 25 µm hatch distance (Figure 4b), the cells showed a less self-organized arrangement but started to visually respect a grid-like pattern, especially with the aligned aspect of the nuclei distribution. However, a noticeable difference was observed (Figure 4e) in the behavior of the cells grown on samples with larger hatch distances compared with the cells grown on sanded bones. Indeed, for the samples with 45 and 65 µm hatch distances (Figure 4c,d, respectively), the cells were mostly contained within the ablated pattern, even though actin filaments and some nuclei could be observed in the area associated with untreated areas. The 3D reconstructions (Figure 4f) provided a more detailed understanding of the cell behavior and highlighted the autofluorescence of the bone imaged with UV wavelengths, merging with the nuclei signals. Looking at the side views of the 3D reconstructions (Figure 4g), higher intensities of actin staining were found within the cavities created via laser ablation, except for the samples patterned with 25 µm, where the signal was diffused. These observations were consistent with those made with regard to the 2D stacks.

**Figure 5:**
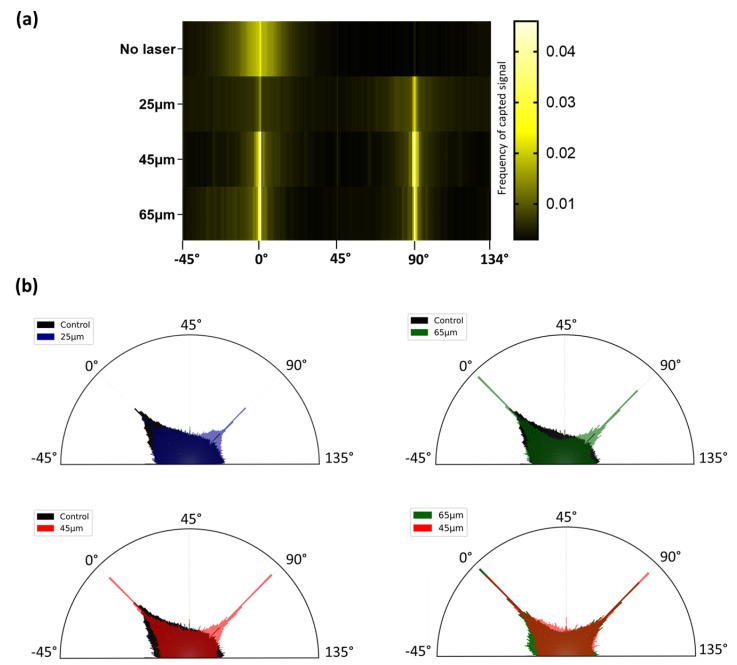
Cell alignment after seeding over laser-patterned samples. Fast Fourier Transform heat map (**a**) highlights the influence of laser patterning on the cell cytoskeletal arrangement, with higher frequencies of aligned cells at 0 and 90°, consistent with the pattern orientations. Cumulated frequency plots (**b**) (proportional to the area of each bar) reveal that frequencies associated with the 45 and 65 µm samples presented overall lower dispersion and higher frequencies at 0 and 90° angles. The control sample was displayed with an opacity of 100%, while all the other conditions had a 50% opacity to be stackable.

The orientation of the actin filaments was analyzed via a Fast Fourier Transform (FFT) analysis of the actin filament channel obtained using confocal microscopy. The retrieved data were analyzed and translated graphically to a heat map (Figure 5a) and frequency plots. On the heat maps, the frequencies are proportional to the intensity of the signal retrieved for each angle. A high frequency indicates a lot of signals from the actin filaments along that axis. The more cells aligned with a given angle, the strongest the signal and the higher the frequency obtained. The heat maps showed that, in the absence of laser patterning, the cells are preferentially oriented in a single range of angles with a single area of rise in frequencies. The laser patterning, being vertical and horizontal, induced two peaks of frequencies, regardless of the hatch size. However, significant differences were observed (Table 2) in the frequencies between 25 µm and the other hatch sizes of around 0°. Around 90°, the h25 vs. h45 mean frequencies were found to be significantly different, and the frequencies from all the test conditions were significantly different from the control.

Frequency plots (Figure 5b) were generated based on the same data. The areas of each bar are proportional to the intensity of the signal for each angle. While the heat maps highlighted the effect of the patterns on dictating the cell adhesion to a defined area, the frequency plots mainly outlined how increasing the hatch distance of the laser-generated pattern resulted in a decrease in the frequencies. The frequencies detected outside the 0° and 90° angles could be assimilated to cells that did not align with the laser-generated pattern: the controls and 25 µm patterned samples were associated with homogeneous dispersion and highest frequencies off the 0 and 90° angles. By increasing the hatch (45 and 65 µm), the frequencies were lowered in the out-of-interest ranges. Yet, these observations were not found to be significant.

Taken together, these results are highly consistent with the aforementioned qualitative results: the cells are mostly aligned with the laser-generated patterns, especially regarding the superior hatch distances of 45 and 65 µm.

### 3.4. Laser Patterning Impact on Cell Adhesion

**Figure 6:**
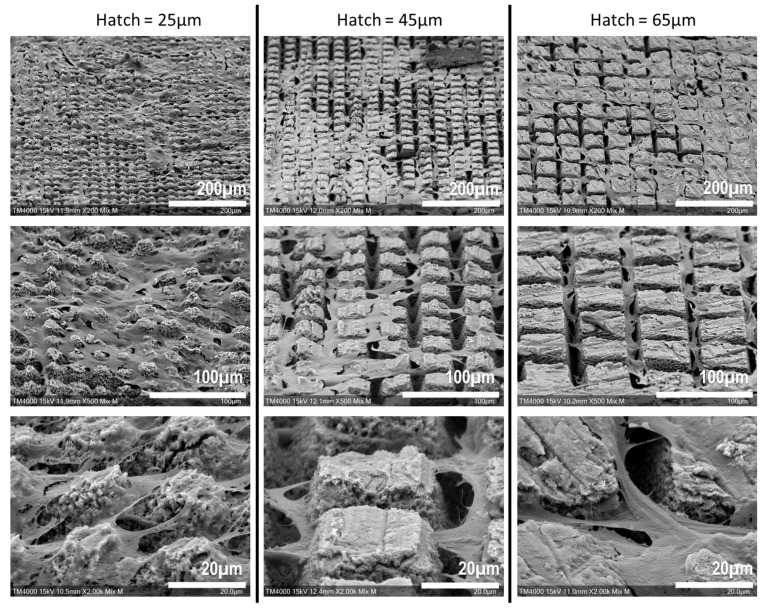
Scanning Electron Microscopy imaging of SCAPs seeded over laser-machined bones. With higher magnifications, the cells appear to adhere to the upper side of the machined patterns and not to the bottom, where the laser hit the most bone surface.

After the confocal imaging, the samples were observed via SEM (Figure 6).

This qualitative assessment of the cell behavior in the machined area revealed that the cells appeared to mostly adhere to the upper side of the machined patterns, while the bottom part of the carved patterns remained unoccupied. However, the cells did not seem to have a preference for adhering to the untreated surface and so fitted to the carved patterns, which is consistent with the confocal microscopy acquisitions.

## 4. Discussion

The results presented in this work on the laser processing of bone tissue demonstrated once again the unique ability of fs laser sources to obtain highly resolutive laser–matter interaction to laser functionalize a biological tissue by precisely tailoring the patterning geometry.

The machining parameters were dictated by two main aspects: designing a fast and reproducible process, and creating a microtextured surface with an impact on the behavior of osteogenic-related cells.

Bone has a highly heterogeneous structure. Here, the samples were collected from the diaphysis part of the femur. The cuboids were extracted from the cortical part of the bone, which is expected to be composed of dense layers with a thickness larger than the sample dimensions [29]. Sanding and patterning steps were applied on the upper surface of the cortical bone (opposite to the medullar cavity). In particular, the sanding step offered the possibility of generating similar surfaces and diminishing the natural topography variation.

The hatch sizes were chosen with consideration of the physiological cell characteristics and behavior. The average circulating MSC cell sizes are between 15 and 30 µm [30]; thus, the influence of the hatch on cell seeding was investigated. A hatch of 25 µm generated surfaces roughly similar to non-patterned bone, meaning that cell settlement was not expected to be influenced beyond the direct effect of laser-induced matter removal. Hatch sizes of 45 µm and 65 µm were associated with the creation of deeper patterns through the cortical bone (about 20 µm deep). It was hypothesized that the cells would preferentially occupy spaces offering three-dimensional anchoring possibilities. The diminution of the carved area proportion within the pattern area was expected either to force the cells to maximally fill the carved trenches, or to create a cell layer above the pattern and secrete extracellular matrix to overcome the gaps between the non-carved areas.

In the experiments, the depth limit ablation was found to be around 20 µm regardless of the hatch size, a quite common effect in fs laser ablation, which could be linked to the partial absorption or shielding of the incoming laser pulse from the laser-generated plasma [31]. This depth limit allowed us to maintain the difference between 45 and 65 µm conditions solely due to the hatch distance discrimination. As seen in the profilometry assay, the ablated area had a conic form, tightening with increased ablation depth. The SEM observations of the upper-adherent cells within the pattern seem consistent with a narrowing of the available space at the bottom of the pattern, rendering it unreachable for large organites such as the nucleus [32], which might lead cells to remain closer to the surface of the pattern.

Among the difficulties encountered, bone autofluorescence made it difficult to properly image the samples. The autofluorescence was corrected with the same thresholding for all the samples to avoid any inter-acquisition bias. Post-acquisition, additional thresholding to remove any residual background noise was associated with the partial loss of cell-related information. Therefore, the images were kept raw and treated under the same procedure for the Fast Fourier Transform. Still facing the difficulty of evaluating the cell viability over time, a metabolic assay was performed to compare the cell activity and give an indirect sight of the cell proliferation through the different patterning evaluated instead of a direct observation such as calcein-AM staining.

SCAPs, a mesenchymal cell population comparable to bone-marrow-derived MSCs [33,34], were used in this study, as these progenitors are more likely to be recruited in situ than mature osteoblasts and are more representative of the diversity of osteoblastic cell populations. The impact of vascular-related cells, such as endothelial cells, should also be investigated in future work.

Laser ablation’s impact on the bone tissue local gene expression profile was previously reported and found to enhance osteogenesis compared with bur drilling [35,36]. However, in this case, all the bones were sanded prior to the laser texturation application to free the mineral matrix from organic ECM and periost. In future work, increased interest should be focused on the transcriptomic consequences of laser patterning.

Although many assays exist to quantify cell adhesion [37], the limitations of working with a thick and opaque material with such a scale of texturation constitute an obstacle for all known standard techniques. As the stated ambition is to improve the bone–material interface strength and quality, further work should be conducted in vivo, inspired by the quantitative assessment of osteointegration by Hallgren et al. [12] and involving histological investigations to investigate the physiological response of laser texturation.

The difficulty of adjusting laser technology from the bench side to the operating room is likely the largest hurdle to its medical application, considering the high cost of a laser compared with other technologies, such as drills or ultrasonication [38,39]. However, this technology is employed with an unprecedented resolution. Moreover, laser patterning could be coupled with laser-assisted bioprinting technology [40], combining the possibility of reshaping bone and then precisely projecting elements of interest (drugs, antibiotics, cells, growth factors, etc.) directly in situ, with the same workstation and a unique laser source.

Some work has been conducted using deep learning to optimize the development of laser machining, predicting the outcome of the skeletal stem cell arrangement according to the laser-machined pattern [41]. The rise of AI-based technologies could significantly improve future research, especially in terms of finding the optimum parameters regarding the patterning design and biological consequences. Future in vivo investigations should be performed with patterns designed to drive cellular colonization with the facilitated settlement of, for example, a vascular network.

## 5. Conclusions

For the first time, laser technology was applied directly to textured cortical bone from the perspective of developing new bone-graft material interface insights for bone tissue engineering. The results showed the high technological potential of femtosecond laser-based processing in tailoring laser-generated bone patterning and its great cytocompatibility with mesenchymal-derived stromal cells. The different patterns investigated revealed the possibility of dictating the cell orientation. Laser patterning was demonstrated to constitute a great opportunity for developing new aspects of the host tissue and graft material interface, and the direct patterning of the host tissue could be considered in future applications of medical devices to enhance grafts.

## Figures and Tables

**Table 1:** Descriptive statistics concerning the bone sample profilometry.

Condition	Measured Hatch ± Std Dev (µm)	Ablation Width ± Std Dev (µm)	Ablation Depth ± Std Dev (µm)	Ablated Area (%)
Control ^1^	N/A	5.37 ± 1.8	4.35 ± 0.99	N/A
H 25	25.61 ± 4.8	8.14 ± 2.7	4.46 ± 1.1	36.6
H 45	45.52 ± 1.9	8.36 ± 1.7	20.06 ± 2.3	26.2
H 65	63.95 ± 1.9	8.5 ± 1.1	21.5 ± 1.1	20.7

^1^ Controls were sanded bone not patterned with a laser.

**Table 2:** Statistical tests over the means of the angle ranges, with a 5° range centered at 0° and 90° matching 5% of the highest value, and the rest of the angles gathering 95% of the lowest values (Kruskal–Wallis, * *p* < 0.05, ** *p* < 0.01, *** *p* < 0.001, ns = not significant, n = 5).

Frequencies	[−45:−3]	[−2:2]	Angle Range [3:87]	[88:92]	[93:134]
Control ^1^ vs. H25	ns	ns	ns	*	ns
Control ^1^ vs. H45	ns	ns	ns	***	ns
Control ^1^ vs. H65	ns	ns	ns	***	ns
H25 vs. H45	ns	**	ns	*	ns
H25 vs. H65	ns	***	ns	ns	ns
H45 vs. H65	ns	ns	ns	ns	ns

^1^ Controls were sanded bone not patterned with laser.

## Data Availability

All data gathered for this article are available from the authors upon reasonable request.
